# Diabetes, Glucose Metabolism, and Glaucoma: The 2005–2008 National Health and Nutrition Examination Survey

**DOI:** 10.1371/journal.pone.0112460

**Published:** 2014-11-13

**Authors:** Di Zhao, Juhee Cho, Myung Hun Kim, David Friedman, Eliseo Guallar

**Affiliations:** 1 Department of Epidemiology and Welch Center for Prevention, Epidemiology, and Clinical Research, Johns Hopkins University Bloomberg School of Public Health, Baltimore, Maryland, United States of America; 2 Department of Health Sciences and Technology, Samsung Advanced Institute for Health Sciences and Technology, Sungkyunkwan University, Seoul, Korea; 3 Saevit Eye Hospital, Goyang, Gyeonggi-do, Korea; 4 Department of Ophthalmology, Johns Hopkins University School of Medicine, Baltimore, Maryland, United States of America; Bascom Palmer Eye Institute, University of Miami School of Medicine;, United States of America

## Abstract

**Background:**

Diabetes may affect vascular autoregulation of the retina and optic nerve and may be associated with an increased risk of glaucoma,but the association of prediabetes, insulin resistance, markers of glucose metabolismwith glaucoma has not beenevaluated in general population samples.

**Objective:**

To examine the relation between diabetes, pre-diabetes, metabolic syndrome and its components and the levels of fasting glucose, HbA1c and HOMA-IR with the prevalence of glaucoma in the general U.S. population.

**Methods:**

Cross-sectional study of 3,299 adult men and women from the 2005–2008 National Health and NutritionExamination Survey (NHANES). The presence of diabetes, prediabetes, the metabolic syndrome and its individual components and biomarkers of glucose metabolisms were based on standardized questionnaire and physical exam data and laboratory tests. The history of glaucoma was assessed through questionnaire during the home interview.

**Results:**

Diabetes was strongly associated with prevalent glaucoma.In fully adjusted models, the odds ratiofor glaucoma comparing participants with diabetes with participants in the reference group with neither pre-diabetes nor diabetes was 2.12 (95% CI: 1.23, 3.67). The corresponding odd ratio comparing participants with pre-diabetes to those in the reference group was 1.01 (95% CI: 0.57, 1.82). Patients with 5 or more years of diabetes duration hadan OR for glaucoma of 3.90 (95% CI: 1.63, 9.32) compared with patients with <5 years of diabetes duration. We also found a hockey-stick shaped associations between biomarkers of glucose metabolisms and the prevalence of glaucoma.

**Conclusions:**

Diabetes was associated with higher risk of glaucoma. Participants without diabetes but at the higher levels of fasting glucose, fasting insulin, HbA1c and HOMA-IR spectrum may also be at greater risk of glaucoma.

## Introduction

Diabetes is a common chronic disease worldwide with a dramatic increase in incidence in recent decades [1]. Diabetes is associated with a variety of ocular complications, including retinopathy, cataracts, uveitis and neovascularization [2]. Several studies also suggested that diabetes may be associated with an increased risk of glaucoma [3]–[5].A meta-analysis of 12 studies published prior to 2004 found a pooled odds ratio for primary open angle glaucoma (POAG) comparing participants with diabetes to those without diabetes of 1.50 (95% confidence interval [CI] 1.16–1.93) [6], but there was significant heterogeneity and many studies reported non-significant associations [7]–[11] or negative point estimates [12]–[15].

Other abnormalities of glucose metabolism, including pre-diabetes, metabolic syndrome, insulin resistance, and elevated fasting glucose or hemoglobin A1c (HbA1c), may also be associated with glaucoma risk, but few studies have examined this issue, with conflicting results [16]–[19].The objective of this study was thus to examine the relation between diabetes, pre-diabetes, metabolic syndrome, and markers of glucose metabolism with the prevalence of glaucoma in the U.S. populationaged 40 years and older using data from the 2005–2008 National Health and Nutrition Examination Survey (NHANES).

## Research Design and Methods

### Study population

The National Health and Nutrition Examination Survey (NHANES) is a nationally representative study of the non-institutionalized US population, obtained by using a stratified multistage probability design with planned oversampling of certain age and minority groups. NHANES is conducted by the National Center of Health Statistics of the Centers for Disease Control and Prevention [20]. Information on glaucoma prevalence was only available in the 2005–2008 survey waves, and fasting glucose and insulin levels were only assessed in participants who were examined in the morning session. Therefore, we restricted our analysis to NHANES 2005–2008 participants 40 years of age or older who were examined in the morning session (N = 3,299). We then excluded 255 participants whose duration of fasting was <8 h (if they didn't have a prior diagnosis of diabetes), 17 participants who had missing information regarding glaucoma in the questionnaire, and 1 participant with missing data in all exposure variables of interest. The final analysis was based on3,026 participants (1,501 men and 1,525 women).

The 2005–2008 NHANES study protocols were approved by the Institutional Review Board of the National Center for Health Statistics. Written informed consent was obtained from all participants.

### Measurements

NHANES included a standardized questionnaire administered at home by a trained interviewer and a detailed physical examination at a mobile examination center. Self-reported glaucoma status was ascertained via the question “Have you ever been told by an eye doctor that you have glaucoma, sometimes called high pressure in your eyes?” Demographic information, education, smoking history, physical activity levels, alcohol consumption, medication use, health history, and age of diabetes onset were also determined by self-report. Leisure-time physical activities were coded and classified according to the rate of energy expenditure (<7.5, 7.5 to <15, and ≥15 metabolic equivalent hours per week) to correspond with cut points in the 2008 US federal physical activity guidelines [21] and the 2010 World Health Organization (WHO) guidelines. [22] Height, weight, and blood pressure were measured at the mobile examination center using standard procedures by trained health technicians [23], [24]. Body mass index (BMI) was calculated as weight in kilograms divided by height in meters squared.

The average (SD) Fasting time was 12.3 (2.6) hours. Fasting blood samples were centrifuged and plasma was separated within 30 minutes from blood collection. Plasma glucose was analyzed using the hexokinase method in a Roche/Hitachi 911 (Roche Diagnostics, 9115 Hague Road, Indianapolis, IN) and a Roche Modular P chemistry analyzer (Roche Diagnostics, 9115 Hague Road, Indianapolis, IN) in 2005–2006 and 2007–2008, respectively [25]. Fasting insulin was analyzed using Merocodia Insulin ELISA kits [20]. HbA1cwas measured using high-performance liquid chromatography on an A1c 2.2 Plus Glycohemoglobin Analyzer (Tosoh Medics, Inc., San Francisco, Ca) in 2005–2006 and on an A1c G7 HPLC Glycohemoglobin Analyzer (Tosoh Medics, Inc., San Francisco, Ca) in 2007–2008 [26].

Diabetes was defined as a fasting plasma glucose level ≥126 mg/dL, an HbA1c level ≥6.5%, a self-reported diagnosis of diabetes (excluding gestational diabetes mellitus), or a self-report of current insulin or diabetes medication use. Pre-diabetes was defined in participants without diabetes as a fasting plasma glucose level between 100 mg/dL and 126 mg/dL, an HbA1c level between 5.7 and 6.5%, or a self-report of a diagnosis of borderline diabetes. Additional analyses were conducted defining diabetes and pre-diabetes using fasting plasma glucose or HbA1 ccriteria separately. Diabetes duration was calculated as the age at interview minus the age at the first time that the participant was told to have diabetes.

Metabolic syndrome components were defined as detailed in the Adult Treatment Panel (ATP) III report [27]: 1) waist circumference ≥102 cm in men and ≥88 cm in women; 2) fasting triglycerides ≥150 mg/dL; 3) HDL cholesterol <40 mg/dL in men and <50 mg/dL in women; 4) blood pressure ≥130/85 mm Hg; and 5) fasting glucose ≥110 mg/dL or use of antidiabetic medication. Persons with at least 3 of these characteristics were defined as having metabolic syndrome. We also calculated the homeostatic model assessment of insulin resistance (HOMA-IR) as fasting glucose (mg/dl) x fasting Insulin (uU/mL)/405 and defined insulin resistance as HOMA-IR >2.6 [28].

### Statistical analysis

All statistical analyses were performed using NHANES weights and *svy* commands in STATA (version 12; Stata Corp., College Station, TX) to account for the complex multistage probability sampling design. Analyses of fasting glucose, fasting insulin, HOMA-IR, and HbA1 clevels were restricted to participants not taking insulin or antidiabetic medications (N = 2,509). Fasting glucose, fasting insulin, HOMA-IR, and HbA1 clevels were categorized into quartiles based on the weighted population distribution.

We used multivariable logistic regression models to calculate odds ratios (OR) and 95% CI for the prevalence of glaucoma associated with glucose metabolism variables. Diabetes and pre-diabetes were included as exposures in the same model, and we fitted separate models for diabetes duration, metabolic syndrome and each its components, insulin resistance, fasting glucose, fasting insulin, HOMA-IR, and HbA1c. For each exposure, we used 3 models with progressive degrees of adjustment. Initial models were crude and then we adjusted for age, sex (male, female) and race/ethnicity (non-Hispanic, white, non-Hispanic black, Mexican American, and other). Finally, fully adjusted models further included education (< high school, high school, > high school), smoking (never, former, current), average physical activity level (low, medium, vigorous), alcohol drinking (<1 drink/week, 1 to <3 drinks/week, ≥3 drinks/week) and BMI (continuous).

For dose-response analyses of the associations of fasting glucose, fasting insulin, HOMA-IR, and HbA1c levels with the prevalence of glaucoma, we calculated OR and 95% CI comparing quartiles 2–4 with the first quartile in categorical analyses, as well as OR and 95% CI comparing the 80^th^ to the 20^th^ percentiles of markers of glucose metabolism modeled as log-transformed continuous variables. In addition, we used restricted cubic spline models with knots at the 10^th^, 50^th^ and 90^th^ percentiles of the distribution of fasting glucose, fasting insulin, HOMA-IR, and HbA1c concentrations to provide a smooth yet flexible description of the shape of dose-response relationship. Tests for non-linear trends computed by log likelihood ratio tests comparing nested models with and without non-linear spline terms.

## Results

The study population had a weighted mean (SE) age of 57.0(0.4) years (**Table 1**). The prevalence of diabetes, pre-diabetes, metabolic syndrome, and insulin resistance were 17.1, 49.0, 32.1, and 43.4%, respectively, and the prevalence of glaucoma was 4.2% (95% CI 3.3–5.3%). The average duration of diabetes was 11.8 years. Participants with prevalent glaucoma were more likely to be older, non-Hispanic black, less educated, and had higher waist circumference, blood pressure, fasting glucose and HbA1c levels (**Table 1**) and to have a significantly higher prevalence of diabetes, elevated blood pressure, and elevated fasting glucose (**Table 2**).

**Table 1:** Characteristics of study participants by glaucoma status.*

		Glaucoma		p-value†
	Overall N = 3,026	No N = 2,835	Yes N = 191	
**Age, year**	57.0 (0.4)	56.5 (0.4)	68.7 (1.5)	<0.001
**Female, %**	53.2 (1.2)	53.2 (1.1)	52.3 (4.9)	0.87
**Race, %**				0.009
** Mexican American**	5.6 (0.8)	5.7 (0.8)	4.5 (1.6)	
** NonHispanic white**	76.2 (2.1)	76.4 (2.1)	71.6 (4.8)	
** NonHispanic black**	10.2 (1.4)	9.8 (1.4)	17.8 (3.7)	
** Others**	8.0 (1.1)	8.1 (1.1)	6.2 (2.5)	
**Education, %**				0.005
** <High school**	18.5 (1.2)	18.0 (1.1)	29.0 (5)	
** High school**	27.0 (1.3)	26.9 (1.3)	30.6 (5.2)	
** >High school**	54.5 (2.1)	55.1 (2.1)	40.4 (4.9)	
**Waist circumference, cm**	100.3 (0.4)	100.2 (0.4)	103 (1.3)	0.04
**Systolic blood pressure, mm Hg**	126.9 (0.5)	126.4 (0.5)	136.3 (2.2)	<0.001
**Diastolic blood pressure, mm Hg**	70.7 (0.4)	70.9 (0.4)	65.7 (1.7)	0.005
**Body mass index, kg/m^2^**	29.0 (0.1)	29.0 (0.1)	29.7 (0.5)	0.15
**Insulin, uU/mL** ‡	11.2 (0.3)	11.2 (0.3)	11.9 (1.1)	0.54
**Serum fasting glucose, mg/dL** ‡	103.5 (0.7)	103.4 (0.7)	108.6 (2.0)	0.01
**HDL, mg/dL**	55.5 (0.3)	55.5 (0.3)	55.4 (1.5)	0.97
**Triglycerides, mg/dL**	146.0 (2.7)	145.6 (2.7)	154.0 (12.7)	0.52
**HbA1c, %** ‡	5.5 (0.02)	5.5 (0.02)	5.7 (0.08)	0.01
**HOMA-IR** ‡	3.0 (0.1)	3.0 (0.1)	3.3 (0.4)	0.40

*Data are means (SEs) or percentages (SEs).

† P value for homogeneity of means or proportions comparing participants with to those without glaucoma.

‡ Values are based on the subsample of participants not taking insulin or medication for diabetes (N = 2,409).

**Table 2:** Prevalence of glucose metabolism abnormalities by glaucoma status.*

		Glaucoma		p-value†
	Overall N = 3,026	No N = 2,835	Yes N = 191	
**Diabetes, %**	17.1 (0.9)	16.1 (0.9)	38.8 (4.1)	<0.001
**Prediabetes, %**	49.0 (1.4)	49.4 (1.4)	40.4 (3.7)	0.34
**Insulin resistance, %**	43.4 (1.5)	43.1 (1.5)	49.8 (4.7)	0.13
**Metabolic syndrome, %**	32.1 (1.2)	31.7 (1.2)	41.1 (5.4)	0.06
** Elevated waist circumference, %**	60.3 (1.4)	60.0 (1.4)	66.4 (4.1)	0.14
** Elevated blood pressure, %**	42.1 (1.1)	41.1 (1.1)	63.3 (4.1)	<0.001
** Elevated triglyceride, %**	33.7 (0.9)	33.7 (1.0)	33.6 (5.5)	0.98
** Reduced HDL, %**	24.0 (0.8)	24.2 (0.8)	18.0 (3.9)	0.17
** Elevated fasting glucose, %**	28.8 (1.4)	28.2 (1.4)	43.3 (3.7)	<0.001
**Duration of diabetes, years**	11.7 (0.6)	11.3 (0.6)	15.2 (1.8)	0.04

*Data are percentages or means (SEs).

† P value for homogeneity of means or proportions comparing participants with to those without glaucoma.

The prevalence of glaucoma in participants with diabetes, with pre-diabetes, and without diabetes or pre-diabetes was 9.5, 3.5, and 2.6%, respectively (P<0.001).In age-, sex-, and race/ethnicity-adjusted models, the OR for glaucoma comparing participants with diabetes to those without diabetes or pre-diabetes was 2.09 (95% CI 1.11 to 3.92) (**Table 3**). The corresponding OR in fully adjusted models was 1.80 (95% CI 0.93 to 3.47). The association of diabetes with glaucoma prevalence was stronger when the definition of diabetes was based on either fasting glucose or HbA1c levels only, with ORs of 2.12 (95% CI 1.23 to 3.67) and 2.10 (95% CI 1.19 to 3.71), respectively. The fully adjusted OR (95% CI) for glaucoma comparing participants with pre-diabetes to those without diabetes or pre-diabetes was 0.88 (95% CI 0.45 to 1.75). Diabetes duration was also significantly associated with glaucoma. Among participants with diabetes, the multivariable-adjusted OR for glaucoma comparing participants with a duration of disease ≥5 years to those with a duration of disease <5 years was3.90 (95% CI 1.63 to 9.32).

**Table 3:** Odds ratio and 95% CIs for the presence of glaucoma.

	Crude model	Model 1†	Model 2‡
**Diabetes**			
** HbA1c or fasting glucose** *	3.99 (2.05, 7.76)	2.09 (1.11, 3.92)	1.80 (0.93, 3.47)
** Fasting glucose only** §	3.83 (2.24, 6.55)	2.28 (1.37, 3.81)	2.12 (1.23, 3.67)
** HbA1c only** †	4.52 (2.65, 7.68)	2.65 (1.49, 4.71)	2.10 (1.19, 3.71)
**Prediabetes**			
** HbA1c or fasting glucose** *	1.36 (0.72, 2.56)	0.96 (0.52, 1.75)	0.88 (0.45, 1.75)
** Fasting glucose only** §	1.23 (0.75, 2.02)	1.02 (0.63, 1.66)	1.01 (0.57, 1.82)
** HbA1c only** †	2.63 (1.53, 4.51)	1.72 (0.98, 3.05)	1.57 (0.85, 2.92)
**Metabolic syndrome**	1.50 (0.98, 2.29)	1.30 (0.83, 2.03)	1.13 (0.60, 2.16)
** Elevated waist circumference**	1.31 (0.91, 1.90)	1.19 (0.81, 1.75)	0.89 (0.55, 1.45)
** Elevated blood pressure**	2.47 (1.71, 3.56)	1.38 (0.97, 1.97)	1.39 (0.92, 2.09)
** Elevated triglyceride**	0.99 (0.59, 1.68)	1.09 (0.65, 1.81)	0.90 (0.52, 1.57)
** Reduced HDL**	0.69 (0.40, 1.18)	0.83 (0.47, 1.47)	0.73 (0.35, 1.52)
** Elevated fasting glucose**	1.95 (1.40, 2.70)	1.35 (1.00, 1.83)	1.29 (0.85, 1.95)
**Diabetes duration >5 years**	2.48 (1.06, 5.76)	2.20 (1.02, 4.73)	3.90 (1.63, 9.32)
**Insulin resistance** ||	1.19 (0.78, 1.81)	1.22 (0.78, 1.90)	1.14 (0.64, 2.01)

† Adjusted for age, gender, and ethnicity.

‡ Further adjusted for smoking, physical activity, alcohol intake, education, and BMI.

*Diabetes defined as self-report, HbA1c ≥6.5%, fasting glucose ≥126 mg/dL, or taking diabetic medications.Pre-diabetes defined as self-report, HbA1c ≥5.7% to <6.5%, or fasting glucose ≥100 mg/dL to <126 mg/dL.

§ Diabetes defined as self-report, HbA1c ≥6.5%, or taking diabetic medications. Pre-diabetes defined as self-report or HbA1c ≥5.7% to <6.5%.

† Diabetes defined as self-report, fasting glucose ≥126 mg/dL or taking diabetic medications. Pre-diabetes defined as self-report or fasting glucose ≥100 mg/dL to <126 mg/dL.

|| Values are based on the subsample of participants not taking insulin or medication for diabetes.

The prevalence of glaucoma in patients with and without metabolic syndrome was 5.3 and 3.6%, respectively (P = 0.06). The OR for glaucoma comparing participants with to those without metabolic syndrome was 1.30 (95% CI 0.83 to 2.03) in age-, sex-, and race/ethnicity-adjusted models and 1.13 (95% CI 0.60 to 2.16) in fully adjusted models (**Table 3**). In fully adjusted models, none of the individual components of the metabolic syndrome or the presence of insulin resistance were significantly associated with the prevalence of glaucoma (**Table 3**).

**Figure 1:**
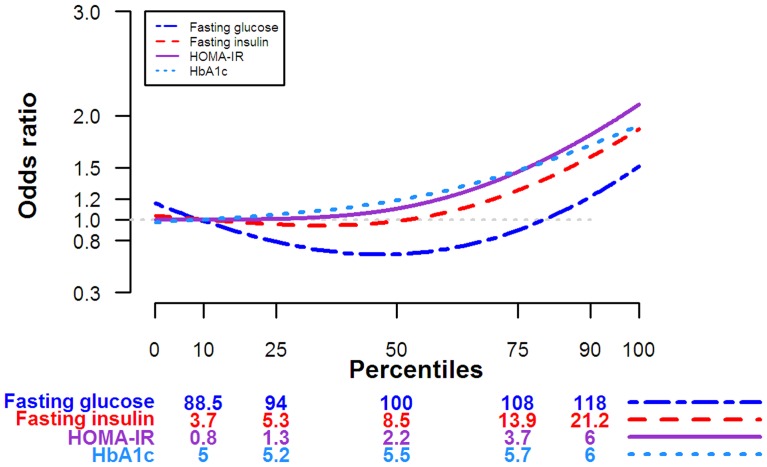
Adjusted odds ratio for glaucoma by levels of markers of glucose metabolism. Adjusted odds ratios were estimated using restricted cubic splines with knots at the 10^th^, 50^th^, and 90^th^ percentiles of the distribution of each marker of glucose metabolism. The reference value (odds ratio  = 1) was set at the 10^th^ percentile of each parameter. Odds ratios were adjusted for age, sex, race, education, smoking status, physical activity, alcohol consumption, and body mass index (see text for details).

In dose-response models with glucose biomarkers modeled as continuous variables or categorized in quartiles, the prevalence of glaucoma increased with increasing biomarker levels but the trends were only statistically significant for fasting glucose (**Table 4**). In fully adjusted models, the OR for glaucoma comparing the fourth to the first quartiles of fasting glucose, insulin, HOMA-IR, and HbA1C were 1.20 (95% CI 0.67 to 2.13; P trend 0.27), 1.22 (95% CI 0.54 to 2.75; P trend 0.75), 1.37 (95% CI 0.57 to 3.27; P trend 0.19) and 1.55 (95% CI 0.59 to 4.08; P trend 0.48), respectively. In spline models, however, the dose-response relationships between markers of glucose metabolism and the prevalence of glaucoma were hockey-stick shaped for insulin, HOMA-IR, and HbA1C and J-shaped for fasting glucose (**Figure 1**).The P values for the non-linear spline terms in the restricted cubic models were significant for all glucose biomarkers (p<0.001 for all).

**Table 4:** Association between markers of glucose metabolism and the presence of glaucoma*

	Odds ratio for glaucoma					P value for quadratic trend
	80^th^ vs. 20^th^	Quartile 1	Quartile 2	Quartile 3	Quartile 4	
**Fasting glucose**						
** Crude Model**	1.37 (1.13, 1.64)	1.00 (reference)	0.71 (0.37, 1.34)	1.06 (0.55, 2.06)	1.56 (0.96, 2.56)	0.02
** Model 1** †	1.22 (0.97, 1.54)	1.00 (reference)	0.67 (0.35, 1.29)	0.98 (0.51, 1.88)	1.05 (0.64, 1.72)	0.47
** Model 2** ‡	1.30 (1.00, 1.68)	1.00 (reference)	0.67 (0.34, 1.34)	1.08 (0.47, 2.47)	1.20 (0.67, 2.13)	0.26
**Fasting Insulin**						
** Crude Model**	1.06 (0.65, 1.73)	1.00 (reference)	1.00 (0.55, 1.81)	0.96 (0.44, 2.06)	1.14 (0.61, 2.15)	0.83
** Model 1** †	1.16 (0.67, 2.01)	1.00 (reference)	1.02 (0.54, 1.90)	0.97 (0.44, 2.11)	1.32 (0.70, 2.49)	0.54
** Model 2** ‡	1.13 (0.53, 2.41)	1.00 (reference)	0.92 (0.43, 1.98)	0.80 (0.30, 2.14)	1.22 (0.54, 2.75)	0.56
**HOMA-IR**						
** Crude Model**	1.16 (0.70, 1.92)	1.00 (reference)	0.67 (0.34, 1.35)	1.36 (0.67, 2.76)	1.15 (0.61, 2.17)	0.24
** Model 1** †	1.21 (0.70, 2.11)	1.00 (reference)	0.68 (0.34, 1.39)	1.39 (0.71, 2.71)	1.22 (0.64, 2.34)	0.17
** Model 2** ‡	1.25 (0.57, 2.72)	1.00 (reference)	0.61 (0.28, 1.34)	1.27 (0.53, 3.05)	1.37 (0.57, 3.27)	0.22
**HbA1C**						
** Crude Model**	1.43 (1.16, 1.77)	1.00 (reference)	1.43 (0.62, 3.31)	2.35 (0.95, 5.85)	3.91 (1.75, 8.71)	0.003
** Model 1** †	1.23 (0.94, 1.60)	1.00 (reference)	1.10 (0.48, 2.51)	1.60 (0.61, 4.21)	1.86 (0.80, 4.28)	0.21
** Model 2** ‡	1.15 (0.85, 1.55)	1.00 (reference)	0.96 (0.38, 2.40)	1.51 (0.53, 4.30)	1.55 (0.59, 4.08)	0.32

*Conducted in people not taking diabetes medications.

† Adjusted for age, gender, and ethnicity.

‡ Further adjusted for smoking, physical activity, alcohol intake, education, and BMI.

## Discussion

In a large sample representative of the general US population, the prevalence of glaucoma was higher in participants with diabetes compared to those with no glucose abnormality, even after controlling for multiple potential confounders. Pre-diabetes and the metabolic syndrome and its components were not consistently associated with the prevalence of glaucoma. However, markers of glucose metabolism showed significant non-linear associations with glaucoma prevalence, including hockey-stick shaped associations for fasting insulin, HbA1c and HOMA-IR, and a J-shaped association for fasting glucose. These non-linear relationships suggest threshold effects for the association of glucose metabolism markers and glaucoma.

The association between diabetes and glaucoma has been evaluated in many studies [9], [13]–[15], [17], [29], [30]. An increased risk of glaucoma in persons with diabetes compared to those who did not have diabetes was also observed in the Beaver Dam Eye study, the Blue Mountains Eye study, the Los Angeles Latino Eye Study and several other population-based studies [3], [5], [16], [19], [31]–[35], but some well-known cohorts did not show statistically significant associations [9], [14], [17]. The discrepancy with these studies may be attributed to study population, sample size, methods to assess diabetes or glaucoma, or drop-out rates. The Baltimore Eye Survey was primarily composed of African Americans and used self-report to define diabetes [9].The Rotterdam Study used a prospective cohort design, but had few participants with diabetes and a high drop-out rate, so that the results were based only on 5 incident cases of glaucoma among 264 participants with diabetes [14].The Singapore Malay Eye Study used random serum glucose measurements instead of fasting glucose to define diabetes, which may result in substantial underdiagnosis, and had also limited power to identify an association between diabetes and glaucoma [17]. In spite of these negative studies, the majority of the epidemiological evidence points to an increased prevalence of glaucoma in patients with diabetes.

Few studies have evaluated the association between metabolic syndrome or glucose metabolism biomarkers and glaucoma, with conflicting results [16]–[19]. In the Singapore Malay Eye Study, participants with metabolic syndrome had a lower prevalence of glaucoma [17], while the number of metabolic syndrome components was positively associated with the hazard of open-angle glaucoma in a US cohort [19]. As for HbA1c and glucose, the Singapore Malay Eye Study showed an elevated but non-significant trend while a case-control study in Europe showed a significantly positive association between increased HbA1c levels and glaucoma [16]. In our study, the association between glucose metabolism biomarkers and the prevalence of glaucoma was non-linear and affected only to participants in the higher half of the distribution of glucose metabolism parameters. These findings suggest that a certain degree of impairment in glucose metabolism is needed before glaucoma appears as a complication of insulin resistance.

In addition to the level of glucose metabolism biomarkers, the duration of the metabolic abnormalities may also be important in determining glaucoma risk. We found that duration of diabetes was significantly associated with an increased prevalence of glaucoma, but we did not have information on the duration of the elevations in glucose metabolism biomarkers or on their trajectories over time. Additional research is needed to confirm these thresholds and to understand the increase in glaucoma risk with increasing duration in glucose metabolism abnormalities.

Several biological mechanisms could explain an increased risk of glaucoma in patients with diabetes. Diabetes may induce structural and functional abnormalities to the small blood vessels feeding the optic nerve, resulting in damage to the optic nerve and the retinal nerve fiber layer [36]. Besides the vascular implications, diabetes may also exacerbate glaucomatous optic neuropathy by increasing the susceptibility of retinal ganglion cells to apoptosis due to elevated intraocular pressure (IOP) [37]. This mechanism may also explain progressive optic nerve injury and neuronal damage with a longer duration of diabetes. Furthermore, diabetic retinopathy may also compromise the glial and neuronal elements, impair retinal function and metabolism, and result in accelerated degeneration of retinal inner neurons [38].

The potential mechanisms underlying the association between glucose metabolism abnormalities and the prevalence of glaucoma in subjects without diabetes are unclear. The presence of the metabolic syndrome and elevated levels of glucose, HOMA-IR and glycosylated hemoglobin may be associated with increased levels of IOP, a key causal factor for glaucoma [31], [39]–[44]. Hyperglycemia increased fibronectin production in the bovine trabecular meshwork, which may increase the resistance to aqueous humor outflow and lead to elevated IOP [45]. Moreover, hyperglycemia could induce apoptosis in retinal neuronal cells through the hexosamine biosynthetic pathway [46]. Additionally, hyperglycemia-induced oxidative stress and advanced glycation end products may increase apoptotic death in retinal neurons [47], [48].

Several limitations need to be considered in the interpretation of our findings. First, the cross-sectional nature of this study limited our ability to establish the causality of the observed associations. Second, while we used high quality laboratory methods and fasting plasma samples to assess glucose metabolism status, the prevalence of glaucoma was assessed by self-report without an ophthalmologic exam. Although the prevalence of glaucoma in NHANES was similar to that reported in other US studies [49], the lack of a clinical assessment of glaucoma may have resulted in underdiagnosis and recall bias. Previous studies of the validity of self-report of glaucoma have shown low sensitivity and very high specificity when compared to eye examinations, and substantial agreement when compared to medical records [50], [51]. Additional studies with high quality measurements of both glucose metabolism parameters and glaucoma status are needed to confirm our findings. Third, the increased prevalence of glaucoma in patients with diabetes and in those with longer duration of diabetes may have been overestimated because of more frequent ophthalmology visits in persons with compared to those without diabetes. We did not have information on the frequency of eye examinations in study participants, but other studies suggested that this type of surveillance bias could not completely account for the positive association between diabetes and glaucoma risk [5], [31]. In the Blue Mountains Eye study, most cases of glaucoma had been diagnosed before the diabetes diagnosis [5], and the Nurses' Health Study also showed that the prospective association between diabetes and glaucoma was unaltered when adjusting for factors related with the number of eye examinations [31]. In spite of these limitations, the large sample size, the standardization and rigorous quality control procedures of NHANES, and the generalizability of the findings to the general US population are important strengths that add to the relevance of our findings.

Glaucoma has a long latency period, in which glaucomatous optic nerve damage is ongoing but remains asymptomatic until later stages. Since vision loss is irreversible, screening and early detection of glaucoma is important in persons with diabetes, given the increased risk for glaucoma and the high frequency coexistence of other mechanisms for vision loss. The adherence to regular ophthalmological exams should be emphasized in diabetic patients, especially among those with long duration of diabetes, regardless of age. Our results also indicate that ophthalmologist referral may be considered for adults without diabetes but with elevated levels of glucose biomarkers.

In conclusion, data from NHANES 2005–2008, a large sample representative of the general US population, showed a higher prevalence of glaucoma in patients with diabetes, particularly in those with longer duration of disease, as well as an increased prevalence of glaucoma with increasing levels of glucose metabolism abnormalities in participants in the higher end of the distribution of glucose metabolism parameters. The mechanisms underlying these associations and the impact of the duration of glucose abnormalities on glaucoma risk need to be established in future studies. Our results support the recommendation that patients with diabetes, as well as those with elevated levels of glucose metabolism parameters, undergo regular ophthalmological exams to monitor for the onset or progression of glaucoma.
